# Exploring Sources of Emotional Distress among People Living with Scleroderma: A Focus Group Study

**DOI:** 10.1371/journal.pone.0152419

**Published:** 2016-03-23

**Authors:** Stephanie T. Gumuchian, Sandra Peláez, Vanessa C. Delisle, Marie-Eve Carrier, Lisa R. Jewett, Ghassan El-Baalbaki, Catherine Fortune, Marie Hudson, Ann Impens, Annett Körner, Jennifer Persmann, Linda Kwakkenbos, Susan J. Bartlett, Brett D. Thombs

**Affiliations:** 1 Department of Psychiatry, McGill University, Montréal, Québec, Canada; 2 Lady Davis Institute for Medical Research, Jewish General Hospital, Montréal, Québec, Canada; 3 Centre de recherche du Centre hospitalier universitaire Sainte-Justine, Montréal, Québec, Canada; 4 Department of Educational and Counselling Psychology, McGill University, Montréal, Québec, Canada; 5 Department of Psychology, Université du Québec à Montréal, Montréal, Québec, Canada; 6 Scleroderma Society of Ontario, Hamilton, Ontario, Canada; 7 Department of Medicine, McGill University, Montréal, Québec, Canada; 8 Institute for Health Innovation, Midwestern University, Downers Grove, Illinois, United States of America; 9 Behavioural Science Institute, Clinical Psychology, Radboud University, Nijmegen, the Netherlands; 10 McGill University Health Center, McGill University, Montréal, Québec, Canada; 11 Department of Epidemiology, Biostatistics, and Occupational Health, McGill University, Montréal, Québec, Canada; 12 Department of Psychology, McGill University, Montréal, Québec, Canada; 13 School of Nursing, McGill University, Montréal, Québec, Canada; University of Texas Health Science Center at Houston, UNITED STATES

## Abstract

**Background:**

Systemic sclerosis, or scleroderma, is a chronic and rare connective tissue disease with negative physical and psychological implications. Sources of emotional distress and the impact they have on the lives of people with scleroderma are not well understood.

**Objectives:**

To gain an in-depth understanding of the emotional experiences and sources of emotional distress for women and men living with scleroderma through focus group discussions.

**Methods:**

Three semi-structured focus group discussions were conducted (two in English, one in French) with a total of 22 people with scleroderma recruited through the Scleroderma Society of Ontario in Hamilton, Ontario and a scleroderma clinic in Montreal, Canada. Interviews were recorded, transcribed, and then coded for emerging themes using thematic inductive analysis.

**Results:**

Core themes representing sources of emotional distress were identified, including: (a) facing a new reality; (b) the daily struggle of living with scleroderma; (c) handling work, employment and general financial burden; (d) changing family roles; (e) social interactions; and (f) navigating the health care system. Collectively, these themes refer to the stressful journey of living with scleroderma including the obstacles faced and the emotional experiences beginning prior to receiving a diagnosis and continuing throughout the participants’ lives.

**Conclusion:**

Scleroderma was portrayed as being an unpredictable and overwhelming disease, resulting in many individuals experiencing multiple sources of emotional distress. Interventions and supportive resources need to be developed to help individuals with scleroderma and people close to them manage and cope with the emotional aspects of the disease.

## Introduction

Systemic sclerosis (SSc), or scleroderma, is a rare, chronic autoimmune disease characterized by fibrosis and internal organ dysfunction including the lungs, heart, and gastrointestinal tract [1]. Symptoms experienced by people with SSc include disfiguring skin thickening, finger ulcers, joint contractures, chronic diarrhea, and renal failure, among others [2]. Common problems experiences by patients with SSc include pain, fatigue, sexual dysfunction, and reduced physical mobility and hand function [3]. The physical and psychological manifestations of SSc are heterogeneous in nature and can have dramatic biological, psychological, and social implications for people living with the disease [2, 4]. A recent Canadian study estimated the prevalence of SSc to be 44 cases per 100,000, including 74 per 100,000 among women and 13 per 100,000 among men [1]. The peak age of onset lies between 40–50 years [1, 4]. Estimated median survival times following diagnosis are 7 years in diffuse SSc, which involves extensive skin involvement and rapid initial disease progression, and 15 years in limited SSc, which involves less extensive skin thickening and a more indolent course [5].

Due to the rarity of the condition, people with SSc face additional challenges compared to those with non-rare conditions, including gaps in knowledge about their disease and how best to treat it [6–8]. Many people with SSc wait long periods of time before receiving an accurate diagnosis or explanation for their symptoms, and many experience difficulty accessing appropriate health care [7, 9]. In addition to medical consequences, people with rare diseases, including SSc, often experience social consequences such as stigmatization, exclusion from social communities, and reduced professional opportunities [7]. As a result, some people with SSc may experience significant isolation as they face life with an unpredictable and potentially fatal disease [7]. Furthermore, supportive resources that are often available to people with common illnesses, such as disease-specific support groups, are often not available or easily accessible for people with rare diseases, including SSc, because of the small number of rare disease patients in any given care center [3].

Studies that have used diagnostic interviews or patient-reported questionnaire data have reported that people with SSc often experience significant emotional distress, including depression, anxiety, fears about disease progression and the future, and body image concerns [10–16]. For example, a Canadian study that assessed 345 SSc patients reported prevalence of major depressive disorder of 4% for the past 30-days, 11% for the past 12-months, and 23% lifetime, which is approximately double the rate in the general Canadian population [16, 17]. Another study, which evaluated 100 French SSc patients found that 49% had at least one current anxiety disorder and that 64% had met criteria for at least one anxiety disorder lifetime [18]. Beyond psychiatric diagnoses, many people with SSc experience significant fear related to disease progression, becoming physically disabled, or becoming dependent on others for help [16, 19, 20]. Body image distress and concerns about appearance are frequent due to changes to highly visible areas of the body, including the face and hands, which can be quite dramatic [16, 21, 22].

Existing quantitative research on emotional distress in SSc has evaluated prevalence, symptom levels, and contributing factors. However, this research has mainly specified *a priori* how distress will be defined and quantified and has not provided participants with the opportunity to freely share their experiences and perspectives concerning the emotional challenges and areas of distress they face [23]. Therefore, research focused on providing people with SSc the opportunity to share their own perspectives is necessary.

We were able to identify five published qualitative studies that have used focus groups or individual interviews to explore the perspectives of people living with SSc [6, 11, 24–26]. Three focus group studies reported major coping challenges for participants and focused mainly on issues related to managing the daily impact of the physical manifestations of the disease [6, 24, 25] or handling the unpredictability of the disease course [6]. A fourth study, which used individual interviews, focused on depression and the label of depression to describe emotional experiences, rather than explore the full range of participants’ emotional experiences [11]. A fifth study, which also used individual interviews, explored the daily life experiences of people living with SSc and identified four main themes, including: (a) the physical impact of the disease, (b) the emotional impact of the disease, (c) the social impact of the disease, and (d) steps taken by participants to cope with the disease [26].

Thus, existing studies have explored the nature of the burden of living with SSc and the distress it causes, but they have not explored, in-depth, the range and impact of the emotional experiences of people living with the disease. Therefore, the objective of the present study was to extend previous research and to develop a comprehensive understanding of both the sources of emotional distress and the nature of the emotional experiences of women and men living with SSc, using focus group interviews. Focus group interviews allow us to obtain information directly from patients and may identify previously unknown experiences that would not be discovered using other common research designs, such as closed-ended questionnaires.

## Methodology

### Research Design and Epistemological Approach

The present study was framed within the social constructionism approach, which is premised on the idea that shared understandings are co-constructed between members of a given social group through the consideration of multiple viewpoints, interactions, exchanges, and through negotiations of meaning [27–29]. Therefore, to foster the interchange among participants and unveil their experiences of enduring similar or unique challenges related to living with SSc, we conducted focus groups.

Focus groups have been widely used in health research, including SSc, to collect data through participants’ discussions and interactions [6, 25, 30–33]. Focus groups are particularly valuable for research when little is known about a phenomenon of interest and gaining a shared understanding of the experiences of participants concerning a specific phenomenon is sought [34].

### Participants and Procedures

We conducted a total of three focus group interviews. Two English-language focus groups were conducted in a hotel conference room in Hamilton, Ontario, Canada, and one French-language focus group was conducted at the Jewish General Hospital in Montreal, Quebec, Canada. Eligible participants were men and women diagnosed with SSc who were fluent in English for the Hamilton focus groups and in French for the Montreal focus group. Potential participants for the English focus groups were members of the Scleroderma Society of Ontario, who were contacted about the study by the society. Potential participants for the French focus groups were enrolled in an ongoing cohort study and contacted by either a research nurse coordinator or a research assistant. Participants who expressed interest in the study were contacted by a study investigator, who provided them with details about the study and set up a date and time for them to participate in one of the three focus groups. Prior to each focus group, additional details about the focus group process were provided, and all participants completed a brief demographic questionnaire in order to record basic demographic and disease information, including age, sex, race/ethnicity, and SSc-related information, such as diagnosis subtype, and years since diagnosis.

The three focus groups ranged in length from 90 to 130 minutes and were conducted between February and July 2013. All three focus groups were held in private meeting rooms and were moderated by a trained psychologist and a graduate-level trainee in psychology. The focus group interviews consisted of a series of open-ended questions aimed at promoting an open discussion about the experiences of emotional distress among people with SSc. After the focus group procedures were explained, the group moderators introduced the topic of emotional distress, asked relevant questions (e.g., With regard to your SSc, what are some things that cause you to experience stress?), and then used probes (e.g., According to you, is the experience you are describing common to all people diagnosed with SSc? Can you elaborate on that?) to encourage a greater sharing of experiences, thoughts, and feelings about certain ideas that arose in the discussions, as well as to gain a clearer understanding of the topic under examination [34, 35] (see S1 Appendix for interview guide). This process of group interviewing allowed the investigator to tease out the strength of participants’ beliefs and subtleties about a topic while capturing individuals’ ideas, experiences, and attitudes as they developed through group interaction and exchange [36, 37].

Due to technical reasons, the two English focus group interviews were recorded by video and audiotape, and the French focus group interview was recorded by audiotape only. All focus group interviews were transcribed verbatim to facilitate future recall and analysis. To better support the presentation of the findings, we extracted participants’ quotes directly from the transcripts. To differentiate the participants, we used F for female and M for male before the numerical identifiers the transcribers arbitrarily attributed to each participant based on the focus group discussion they were in and the order in which they first spoke while participating within their group. The community of Canadians with SSc is limited in size. Thus, in order to protect the confidentiality of the participants, we purposefully did not present individual demographic characteristics in the present manuscript, that could serve to identify the participants.

This study was approved by the Research Ethics Committee of the Jewish General Hospital in Montreal, Quebec, and all participants provided written informed consent. Following each focus group, participants were reimbursed $20 for travel costs.

### Data Analysis

A thematic analysis approach [38] was used to analyze data. Thematic analysis is a qualitative research method that supports the identification of new ideas directly from the data, while also relying on existing literature to help integrate novel and predetermined ideas. Thus, this analytic approach allowed the investigators to openly explore the participants’ viewpoints and at the same time, to enrich our knowledge and understanding of the phenomenon of interest [38–40].

A professional transcriber fluent in English and French was responsible for the transcription of the three focus groups. One investigator independently initially analyzed the transcriptions of the two English focus group interviews, and a different bilingual investigator initially analyzed the transcriptions of the French focus group interview [41]. These investigators followed the same analysis steps, as follows: First, the investigators read the transcriptions several times to achieve full immersion in the text data and to obtain a sense of it as a whole. Second, different fragments of the text representing participants’ discussions were assigned a code depending on their meaning. Third, based on comparisons among different coded groups, similar codes were grouped together. The investigators then created a preliminary coding scheme based on potential relationships among codes. As such, themes and sub-themes were identified. To assure the consistency of this preliminary coding scheme, the investigators used it to recode the three transcriptions in full. Following this, the investigators re-read the document and highlighted quotations that appeared to capture key thoughts or concepts that meaningfully expressed the core idea of each theme.

Following the development of a preliminary coding scheme, a second bilingual investigator thoroughly reviewed all three coded documents. Then, the investigators discussed their coding in order to achieve consensus upon codes and to resolve any discrepancies in text codings. As a result of these discussions, a sustainable coding scheme was developed. No differences were identified when between-group comparisons were conducted.

To illustrate the themes, we extracted quotes from the transcripts. All patients contributed to the focus group discussions; but, to represent each theme, we chose the most representative and compelling quotes.

As suggested by Poland [42], after agreeing in the meaning of selected quotes, we corrected and adjusted conversational mechanic errors (e.g., incorrect grammar use). Data analysis was supported by the use of the qualitative research software *Atlas*.*ti* [43].

## Results

### Participant Characteristics

A total of 22 individuals with SSc (18 females, 4 males) participated in one of the three focus groups (1^st^ English = 6 females; 2^nd^ English = 4 females, 1 male; French = 8 females, 3 males). Sociodemographic characteristics are presented in Table 1. The age of participants ranged from 26 to 77 years with a mean age of 53.3 years (Standard Deviation [SD] = 13.3). Of the 22 participants, there were 2 (9%) who reported that they had been diagnosed with limited SSc, 10 (45%) with diffuse SSc, 5 (23%) with CREST, 5 (23%) who were unsure of their disease subtype. Years since diagnosis ranged from 0 to 28 with a mean of 10 years (SD = 8.0). Of the 22 participants, the majority were White (86%), had completed at least some college education (77%), and were either retired (23%) or on disability leave (32%).

**Table 1:** Sociodemographic Characteristics of 22 Focus Group Participants.

Variable	
Female gender, *n (%)*	18 (82%)
Age in years, *mean (SD)*	53.3 (13.3)
White race/ethnicity, *n (%)*	19 (86%)
Level of education, *n (%)*	
Less than high school	1 (5%)
High school graduate	4 (18%)
College/CEGEP^a^ graduate	7 (32%)
University graduate	8 (36%)
At least some postgraduate	2 (9%)
Occupational status, *n (%)*	
Homemaker	3 (14%)
Unemployed	2 (9%)
Retired	5 (23%)
On disability leave	7 (32%)
On leave of absence	1 (5%)
Full-time employed	2 (9%)
Part-time employed	2 (9%)
Scleroderma subtype, *n (%)*	
Limited scleroderma	2 (9%)
Diffuse scleroderma	10 (45%)
CREST	5 (23%)
Unsure	5 (23%)
Years since diagnosis, *mean (SD)*	10 (8.0)
Years since Raynaud’s onset, *mean (SD)*	21 (13.3)

^a^Collège d'enseignement général et professionnel (CEGEP) is the post-secondary degree equivalent to grade 12 of high school and first year of university in the province of Quebec, Canada.

### Participants’ Perspectives Concerning Sources of Emotional Distress

Six overarching themes related to perceived sources of distress, as described by study participants, were identified: (a) facing a new reality; (b) the daily struggle of living with scleroderma; (c) handling work, employment and general financial burden; (d) changing family roles; (e) social interactions; and (f) navigating the health care system. Code definitions and the coding scheme are provided in the S2 Appendix.

#### Facing a new reality

The process of receiving a diagnosis, according to participants, entailed two different stressful events. First, some participants explained that, for a period of time, they endured a multitude of symptoms before being properly diagnosed. Participant M-P11 said, *“my family doctor […] tested me and my blood*, *said that I carried lupus*, *and further on they’re like no that’s not what it is*. *And it took*, *like I said*, *it took a long time to narrow it down*.*”*

Coming to terms with the actual diagnosis of SSc was another emotionally challenging experience due to the unpredictable nature of the disease. For many participants, a major source of emotional distress after receiving a diagnosis related to not knowing how the disease might progress. Participant F-P4 explained, *“after the first year I just fell into a major depressive episode where I was just in bed for like 7 months and that was it*.*”* The same participant said, *“the most frustrating thing about the disease is that once you get to a point where you’re comfortable with the level of symptoms and interactions you have*, *then all of a sudden it progresses and then something else goes wrong*.*”* Unpredictability was also related to the knowledge that there is no cure for SSc, which was especially difficult for some participants. As participant M-P11 described, *“the hardest part for me was getting my head around the diagnosis*. *You’re not going to be off these drugs*, *you know*, *[and] as of right now it’s not going to get better*.*”*

Ultimately, this uncertainty about what the future might hold resulted in participants voicing concerns about seeing others with SSc in worse condition. As a result of this, many participants struggled with the decision of whether or not to attend SSc conferences or support groups as they often felt discomfort over the idea of seeing someone with SSc in worse condition than themselves. Participant F-P8 explained, *“the most difficult part was to go to something for the first time*, *where there would be people with scleroderma*, *because I was afraid of what I would see*.*”*

#### The daily struggle of living with SSc

Two major burdens arose within the day-to-day lives of participants, including dealing with symptoms of the disease and undergoing treatments. Participants explained the hardships, frustrations and feelings of hopelessness that arise on a daily basis as they experience and attempt to manage psychological symptoms, such as feelings of depression; and physical symptoms, such as Raynaud’s phenomenon, gastrointestinal complications, and breathing problems, simultaneously. Participant F-P1 described experiencing multiple symptoms as *“having your 20*^*th*^ *kid and trying to think how are you going to feed that one [when] you’ve got another one coming along next year you’ve got to deal with”* and that *“it grows*, *it’s like a cancer; it’s going out of control*.*”* In this vein, Participant F-P1 described, *“I can’t cope with continual pain that just will not go away*. *I can’t function*, *I can’t do anything*.*”*

For these participants, experiencing fatigue and having problems sleeping made it impossible to consistently meet daily demands, such as keeping their house clean, finishing home renovations, or grocery shopping. Participant F-P4 explained, *“with scleroderma you literally have a bank account of let’s just say 1,000 points of energy*, *but you need*, *I don’t know 20,000 points of energy to get what you need to get done*.*”*

Additionally, many participants experienced feelings of guilt when their symptoms did not permit them to meet expectations that others had for them. Participant F-P9 described, *“it’s very hard for everybody else to perceive how hard it is for you to manage through the day*. *I feel guilty because people have expectations*. *And*, *well your house should be clean*. *Well my house is not clean by any means anymore*. *And that’s guilt—Oh*! *I should pick this up or I should pick that up*. *But for me as soon as I go to bend over*, *I have regurgitation immediately*.*”* Ultimately, this inability to complete daily tasks resulted in some participants changing their priorities and realizing that *“whatever was really*, *really important that you had to do*, *all of a sudden [doesn’t] matter as much anymore”* (F-P10).

Facing the reality of ongoing appearance changes was also discussed as being a significant daily stressor. Participant F-P9 explained, *“We had family pictures done at Christmas and I couldn’t pick out our pictures that had me in it because I didn’t look the way I remember looking…*.*my husband actually had to pick out all the pictures because all I could see were all the changes that happened over the last 4 years*.*”*

Experiencing depressive feelings, as well as uncertainty, fear, and anger was common among group members. In this vein, participant F-P2 explained, *“the next day you wake up and you feel really good and all of a sudden those tears start*, *and you think*, *holy crap*, *like what’s wrong with you*? *Am I depressed or not*?*”* In some cases, the distress and the depressive feelings were difficult to deal with and had an important negative influence on participants’ personal lives, as participant F-P7 described, *“I allowed my depression to get involved with my marriage”* ultimately resulting in its *“breakdown*.*”*

Manifestations of SSc typically include Raynaud’s phenomenon, in which cold temperatures or other triggers lead to narrowing of the blood vessels, discoloration, and pain in extremities. As a result, managing and adjusting to cold temperatures or temperature changes was another area identified as being significantly challenging and frustrating for many of the participants on a regular basis. Participant F-P3 illustrated this problem by explaining that while shopping, *“I found a couple of things I wanted to try on*. *Well I don’t try things on*, *like I don’t have that luxury*. *I mean I can buy something and return it but it’s freezing in the room*.*”* Participants also agreed that it was especially upsetting when they were no longer able to participate in the same activities as before their SSc symptoms intensified due to the cold. Participant M-P11 explained, *“I don’t even go ice fishing; I don’t even go to the cottage in the winter*. *I just can’t handle the cold*, *you know*, *like 5 minutes I’m done*. *I’m absolutely done*.*”*

Undergoing various treatments, medical procedures, and experimenting with different medications were identified as being the second main emotional burden within the daily lives of participants. Participant F-P7 described *“the problem with scleroderma is that there’s no treatment; no drug [can] help [every] person*.*”* Not only were participants frustrated with having to take multiple pills without always experiencing significant improvements in their symptoms, but many also struggled to cope with the side-effects of the different medications they were prescribed. For participant F-P4 *“it got to the point where you’re just…*.*for me over the summer when they switched my medication and I just kept constantly throwing up and saying*, *‘It’s not working*, *I’m not functioning*.*’ to the point where you just say*, *‘I’m not taking medication anymore because I got so fed up*, *like nothing would work’*.*”* Through tears, participant F-P3 described her infusion treatments for Raynaud’s phenomenon as: *“terrifying*, *it was beyond painful how symptomatic I was to this infusion*. *I’d never experienced that in my life*.*”*

#### Managing work, employment, and general financial burden

Participants agreed that living with SSc dramatically impacted their ability to work, and as a result many participants have had to stop working or adopt a part time schedule. Participant F-P10 explained, *“I did work full-time*, *I was a hard worker*, *carried on 2 full-time jobs at one point and now I’m just a part-time worker*. *That’s all I can do right now*.*”* Additional frustrations arose when participants discussed dealing with their employers. Participant M-P11 described, *“I still work full-time*, *I work shifts; fatigue has become a real issue*. *It’s unfortunate my employer has not been of any help at all; I thought they would have been*. *I’ve requested to reduce my shift work and some other things*, *and it’s almost like*, *they seem like it’s not even a recognized issue that I am going through*.*”*

The inability to work translated into concerns about affording health care, medications, and specialist appointments, as well as having to buy additional items (e.g., gloves, warmer coats), to help participants cope with and manage the disease. In Canada, basic medical care is covered universally; however, prescription medications and other alternative treatments commonly required by people with SSc are typically not covered, resulting in many people the disease having to pay out of pocket in order to receive the care and treatments they need. Participant F-P1 expressed, *“it’s all revolved about money*. *Money will help you be better or you’ve got to buy all these drugs*. *And you don’t have it*. *And that is a major stress because it never sinks in […] that you’re financially strapped*.*”* Participant F-P1 described, *“the impact in terms of stress that the loss of your job and loss of your income is*, *if you’re married of course your income now is minus the income of the female*. *And I say that because it’s more females than males that get scleroderma*. *But for single people*, *it’s having an income to having zero*.*”*

#### Changing family roles

Many participants spoke of their families as being positive and supportive, however some reported that discussing aspects of living with SSc with family members and partners was difficult and stressful. It became clear that some people felt their family members *“forget you actually have something wrong with you”* (F-P2). According to participants, sometimes their family members do not realize the extent to which they experience symptoms or struggle with their SSc, resulting in comments such as, *“you don’t look sick”* (F-P4). Participant F-P3 explained the importance of being able to communicate with family members and partners about symptoms as, *“people can’t see the fatigue*, *stress*, *pain*, *and the sleep deprivation […] so you’ve got to tell them*.*”*

Many participants experienced feelings of guilt and worry when they felt they were letting their families down by not being able to maintain their roles as the primary caregivers within their homes. Participant F-P9 discussed her regrets, *“my two older children had the best of me*. *We were at the park all the time*, *we were sledding*, *we were skating*, *we were everything always*. *My little 5 year old did not get that [at] all*.*”* Participant F-P9 admitted, *“I’m always trying to solve it for my kids to make it easy for them and for the people around me*. *Because this is difficult for me to handle and not that I’m the matriarch*, *but my kids will tell you I’m the glue that holds the family together*, *so when I go into the hospital for five days*, *everybody literally falls apart when I’m not there*. *So I feel like I have to be that strong person*.*”*

Other participants’ felt like having SSc made them a burden to their families. Participant F-P4 explained, *“when the disease first came about*, *my whole family blamed me for having the disease just because they’re super religious and they thought God was punishing me*.*”* As a result, participant F-P4 said, *“[I] can’t talk to my family about my disease because they throw it back in my face*. *So that’s my number one rule*, *don’t talk about my health with my family which makes it difficult because they’re family and I’m looking for support*.*”* In a similar vein, participant F-P10 admitted, *“I don’t want to be a burden on people*, *so I haven’t told my family things*.*”*

#### Social interactions

Social interactions beyond the family, were also identified as a source of emotional distress because many participants felt exposed by their visible differences and, as a result, received unwanted attention. As participant F-P3 explained, *“I was in the grocery store*, *I went into the freezer section and you know had the […] same old story*, *and really painful*. *And now I’m losing function of my hands and I can’t get my coins out of my purse […] and I could feel the anxiety level growing*. *And a woman you know was very [annoyed]*, *like these are just strangers that feel a need to just comment*, *and I got so upset*.*”* Participant F-P4 discussed receiving *“the same comments when I’m wearing a sweater or a tuque in the middle of summer*, *it’s like it’s not that cold*, *it’s just like you have no idea*.*”*

Participants were also frustrated when others did not understand the severity of the disease as all they see is a *“perfectly*, *healthy*, *working individual”* (F-P1). Participant F-P9 described their experience, *“in the summertime my mother-in-law was telling family about how sick I had been*. *And my husband and I took the kids to go visit these people*. *And I walked in the door and they were flabbergasted*, *‘You look so good*, *you look wonderful*!*’ From all the horror stories they had heard*, *they had expected me to be dragging myself in*. *And so it makes it hard for them to believe that you’re really sick*.*”* Participant F-P9 admitted, *“you know what my mom always says when you go to the doctors*? *Don’t put on any makeup*, *make sure you’re looking all worn so that it’s more believable*. *And you know I said*, *I said to my mom*, *‘Why should I have to fake it*?*’ Right*? *I really am that sick*.*”*

Participants also felt frustrated and angry knowing that many people labeled or identified them as the *“individual with scleroderma*.*”* Participants F-P2 and F-P3 admitted that they *“don’t want [scleroderma] to be [their] identifier*.*”* This frustration was extended when participants felt they were being given special treatment or receiving pity from their families, friends, or others, because of the disease. In participant F-P9’s experience, *“there are some family members who try to butt in and want to take over some of the things that I do*, *and then there’s resentment there because they can do it and it’s so hard for me to do it*.*”* As participant F-P8 discussed, *“nothing was more annoying than somebody wanting to come up and open the can of tuna fish for me because I was struggling*.*”*

#### Navigating the health care system

The last source of emotional distress discussed by participants was the health care system, and more specifically, communication with health care professionals and having too many medical appointments. Participant F-P4 explained, *“I was telling [my doctor] about how my symptoms were worsening because I had stomach emptying problems to the point where I couldn’t eat anything*. *Anything I ate it would just come right back up*. *I had no energy*. *I was having trouble breathing*. *Lots of problems over the summer*. *And*, *when I was telling him all the problems I was having and the services that I needed*, *he said*, *‘Too bad*, *so sad’*.*”* Participant F-P3 described her experiences with the medical community as being *“dismissive”* as she felt that *“when another body part starts to ache”* doctors *“automatically assume its scleroderma related”* and therefore they do not give her enough time to thoroughly examine her symptoms and provide her with appropriate explanations. When discussing her specialist, participant F-P3 explained, *“I see mine every three months*, *but you know we’ve got a set parameter of things that we discuss and very limited time*.*”*

Participants also expressed frustration with the amount of specialist referrals and appointments they had. For example, participant F-P9 reported having *“seven specialists”* (F-P9). Participant F-P10 explained, *“this is because we keep getting referred*. *Here’s another specialist*, *here’s this*, *here’s that*.*”* When asked about how these appointments directly caused stress in the participants lives, participant F-P10 explained, *“well I’ve got to take time off work; I’ve got to go here*, *got to go there; got to take their medicine for this”* to which participant M-P11 agreed, *“I just can’t keep taking time*, *days off*, *and using up all my vacation days or my ten government personal days every time I’ve got to go*, *but at the same point they’re specialists and it’s kind of like okay well…”*

Another area causing distress among participants concerns obtaining accurate and helpful information from health care personnel about issues related to intimacy and sexuality with SSc. Participants discussed that information covering aspects of sexuality with SSc is scant, which was particularly discouraging for some participants. Participant F-P9 describes her experience when asking her doctor about ways to address intimacy concerns as follows: *“we went to the Doctor and said*, *‘Hey give us a hint here*, *there’s a real problem*.*’ And you know what he said*? *He told my husband*, *‘You let her relax for the day and you take care of everything and maybe something can happen*.*’ That was their advice*. *And it’s like*, *‘Are you kidding me*?*’*.*”*

Lastly, participants expressed great anger and frustration when discussing their health coverage and services, as many felt they were not provided with adequate care or had limited access to satisfactory health services. Participant F-P4 felt that her organization was withholding services because *“they thought [she] wasn’t sick enough”*. She expressed her frustrations with health services: *“does he not understand how severe my disease is and how specific my dietary requirements are and things that I need to manage daily*? *And when I told him that*, *you know*, *I was throwing up and sometimes it would end up on the floor or all over my bathroom and that the PSW [personal support worker] said that they couldn’t enter my house it’s a dangerous environment*. *Obviously like that doesn’t make any sense because if I can’t clean it up because of my energy or I can’t bend down or manage water*, *then what do I do*? *And he didn’t have an answer for me*.*”*

## Discussion

The main finding of this study was that sources of emotional distress for people with SSc are multifaceted in nature, begin prior to receiving a diagnosis, and continue throughout their lives in an unpredictable manner. Six main themes related to sources of emotional distress were identified, including: (a) facing a new reality; (b) the daily struggle of living with scleroderma; (c) handling work, employment and general financial burden; (d) changing family roles; (e) social interactions; and (f) navigating the health care system.

Of the six themes identified in the present study, three were consistently reported in all five previous studies [6, 11, 24–26] that explored emotional distress among people with SSc using qualitative methods: Facing a New Reality, the Daily Struggle of Living with SSc, and Social Interactions. In all five studies, participants discussed issues consistent with Facing a New Reality theme, including the process of receiving a diagnosis, experiencing uncertainty about the progression of the disease, and developing feelings of anxiety about the future. Concerning the Daily Struggle of Living with SSc theme, participants in previous studies similarly reported stress due to experiencing and managing the physical and emotional symptoms of SSc, learning how to manage cold temperatures, experiencing treatment and drug side effects, and maintaining everyday responsibilities including chores and general housework [6, 11, 24–26].

Three of the themes reflecting experiences of sources of distress identified in the present study were not reported in depth in previous qualitative studies: Handling Work, Employment, and Financial Burden; Changing Family Roles as a Potential Source of Stress; and Navigating the Health Care System. Related to Handling Work, Employment, and Financial Burden, participants described the emotional burden related to modifications that they had to make to their occupational or professional roles because of their illness. Many participants discussed having to quit their jobs or reduce the amount of hours they could work. In turn, these reductions in income resulted in participants feeling financially stressed and burdened as many struggled to afford the drugs, treatments, and additional resources needed to be able to effectively cope with and manage their SSc.

The second novel theme was Changing Family Roles. In past research, family members of people with SSc were generally discussed in a positive light, with the family providing support and additional help to the individual with SSc [11, 24, 26]. However, in addition to these positive aspects, many participants in the present study, which focused on stressors, discussed families as an additional source of distress in their lives. Some participants reported feeling overwhelmed and frustrated when discussing their SSc diagnosis with their family members, and some felt they had become burdens to their families as they could no longer maintain their typical roles and responsibilities within their households.

The last unique theme identified and explored in this study concerns the participants’ stressful experiences attempting to navigate the health care system. Although this concept has been mentioned previously [24], the extent to which navigating and dealing with the health care system can cause stress among people with SSc is not well understood. Participants in the present study identified several aspects of their experiences with the health care system as causing significant stress, including having multiple specialists and appointments, experiencing the overgeneralization and dismissive nature of patient-doctor communication, as well as other general problems concerning the organization of the health care system.

Although SSc is a rare disease, some of the sources of emotional distress identified in the present study are also experienced by people with more common chronic diseases, such as cancer and arthritis [6, 11]. Other sources of distress, however, such as difficulty obtaining an accurate diagnosis and specific aspects related to navigating a health care system not designed for people with rare diseases may be unique to the rare disease context [7, 8]. It is common for people with rare diseases to be improperly diagnosed or have to wait long periods of time before receiving an accurate diagnosis [6, 8]. Additionally, people with rare, disfiguring diseases may face additional social consequences such as isolation and stigmatization, compared to those with more common and invisible chronic illnesses. Due to the limited amount of available rare disease-related resources it is not uncommon for people with rare diseases to have to wait long periods of time or travel long distances in order to receive appropriate care and support. For many rare diseases, a lack of scientific knowledge and available information about the disease or how to treat it can make navigating the health care system particularly difficult and lead to having to consult with multiple doctors and specialists simultaneously. This process not only causes financial stress, but can be particularly confusing and exhausting for people with rare diseases, as participants with SSc described within the present study.

Supportive resources to help people with SSc cope with the sources of distress identified in the present study need to be developed. To date, the majority of research on treatments for SSc focus on the management of medical symptoms (e.g., gastrointestinal disease) and on improvement of physical functioning. Findings from the present study and previous studies [6, 11, 24–26] emphasize that people with SSc experience a significant amount of emotional distress related to their disease, some of which may be specific to people with rare diseases.

Information gathered from the present study will be highly useful to inform the development of resources designed specifically for people with SSc, to help them cope with and manage the six identified areas causing them to experience disease-related emotional distress. Specifically, the Scleroderma Patient-centered Intervention Network (SPIN) [3, 44], an international collaboration of SSc patient organizations, clinicians, and researchers, was formed to develop supportive resources aimed at improving health-related quality of life outcomes among people with SSc. Thus, SPIN will use the information gathered from the present study to develop online supportive resources and tools that can be used by people with SSc to help them learn how to better manage and cope with the emotional distress they face. SPIN has chosen to develop these resources in an online format, in order to reach as many people with SSc as possible, especially patients who live in rural areas far from specialized care who are often left to cope with their disease alone as well as for patients who are concerned about social situations or seeing others with SSc in worse condition than themselves.

There are a number of limitations that should be considered when interpreting the results of this study. First, individuals who participated in this study constitute a convenience sample of SSc patients. Specifically, recruitment occurred through the Scleroderma Society of Ontario and through a research nurse coordinator research assistant at a single hospital site, which may have influenced the characteristics of respondents, and therefore may limit the generalizability of conclusions about the identified areas of distress for people with SSc in other geographical locations. This is because people with SSc who are able to participate in these studies may be in healthier conditions than those who cannot, therefore our results might be an underestimation of the emotionally distressing experiences faced by people with SSc. Secondly, male and female participants were combined in the present study, and it is possible that the sources of emotional distress differ between the sexes. Only a small number of men were included, and it is possible that the focus group format may not have permitted the identification of stressors specific to men living with SSc. For instance, although we did not have any direct evidence for this, it is possible that some of the male participants may not have felt comfortable disclosing information about their emotional experiences within a female-majority group setting. Third, both the number of participants in each of the three focus groups as well as the facilitators of these groups differed between groups. Because the focus group interviews were semi-structured and led by different researchers, participants within the three groups may not have received precisely the same original research questions or probes. Fourth, the English focus groups were recorded by videotape, whereas the French focus group was recorded by audiotape only. It is possible that there could have been differences between the groups, for instance, in the extent to which participants felt comfortable freely discussing their experiences, because of this difference. Lastly, the participants in each of the three focus groups were only interviewed once, limiting the opportunity for all the participants in the group to have ample time to express their personal experiences. There is a need for future studies that examine the emotional experiences of SSc among men specifically. Although SSc predominantly affects females, men with SSc may experience unique emotional consequences that are currently not well understood.

## Conclusion

In conclusion, there are many different aspects of living with SSc that may cause people with the disease to experience emotional distress. The present study identified six core themes related to sources of distress, including: (a) facing a new reality; (b) the daily struggle of living with scleroderma; (c) handling work, employment and general financial burden; (d) changing family roles; (e) social interactions; and (f) navigating the health care system. These six themes represent the main sources of distress experienced by a group of people with SSc. The findings of this study provide insight into the unique emotional experiences and challenges faced by people living with the rare and unpredictable disease, SSc. These findings will be used by SPIN to inform the development of online supportive resources and tools aimed at improving quality of life and coping among people with SSc.

## Supporting Information

S1 Appendix Interview Guide (Adapted to Each Participant Group). (DOCX)

S2 Appendix Identified Themes, Sub-themes, and Corresponding Definitions. (DOCX)
