# Supplementary material for: Exploring Sources of Emotional Distress among People Living with Scleroderma: A Focus Group Study
Source: PLoS One. 2016 Mar 23;11(3):e0152419. doi: 10.1371/journal.pone.0152419 (PMC4805283; doi:10.1371/journal.pone.0152419)
Supplement: S1 Appendix — (DOCX) [file pone.0152419.s001.docx]

**S1 Appendix. Interview Guide (adapted to each participant group)**

**Overview:**

These are examples of some of the initial questions posed to participants to initiate the discussion. Further questions and probes were used to support the conversation as it progressed naturally.

1. **What are some areas of living with SSc causing you emotional distress?**
   1. What term(s) would you use to describe your emotional experiences related to living with SSc?
   2. Explain to me how these areas you have identified as being distressful cause you to experience disease-related stress?
2. **What are some of the different emotions you have experienced while living with SSc?**
   1. What areas of living with the disease have made you experience anger of frustration?
   2. Are there any emotions we have not covered during this discussion that you experience or have experienced while living with SSc and you would like to share?
