# Supplementary material for: Exploring Sources of Emotional Distress among People Living with Scleroderma: A Focus Group Study
Source: PLoS One. 2016 Mar 23;11(3):e0152419. doi: 10.1371/journal.pone.0152419 (PMC4805283; doi:10.1371/journal.pone.0152419)
Supplement: S2 Appendix — (DOCX) [file pone.0152419.s002.docx]

**S2 Appendix. Identified Themes, Sub-themes, and Corresponding Definitions**

| **Theme and Subthemes** | **Codes** | **Definitions** |
| --- | --- | --- |
| **Facing a New Reality** | Difficulty with diagnosis | Experiencing distress due to not receiving a timely and accurate diagnosis. |
|  | Receiving a SSc diagnosis | Experiencing distress from the process of receiving a SSc diagnosis. |
|  | Uncertainty about progression of disease | Experiencing distress as a result of feeling uncertain about the progression of their SSc. |
|  | Seeing someone in worse condition | Experiencing distress as a result of seeing someone with SSc in worse condition than themselves. |
| **The Daily Struggle of Living with SSc** |  |  |
| Experiencing and managing psychosomatic symptoms | Comorbidity of symptoms | Experiencing distress caused by having multiple physical or psychological symptoms at the same time. |
|  | Dealing with unique symptoms of SSc | Experiencing distress caused by experiencing unique symptoms or situations caused by their SSc. |
|  | Psychological symptoms | Experiencing distress because of psychological factors or due to having symptoms of depression or not understanding depressive feelings. |
|  | Physiological symptoms | Experiencing distress caused from having constant pain, fatigue, appearance changes, Raynaud’s Phenomenon, tight skin, gastrointestinal symptoms, and breathing problems. |
|  | Dealing with the cold and temperature changes | Experiencing distress caused by experiencing cold temperatures or temperature changes. |
|  | Doing general housework | Experiencing distress due to having to do housework. |
| Treatment complications | Experimenting with medications | Experiencing distress as a result of trying new medications or experiencing medication side-effects. |
|  | Undergoing treatments | Experiencing distress as a result of undergoing various treatments for SSc, including side effects. |
| **Handling Work, Employment, and General Financial Burden** | Being unable to work | Experiencing distress as a result of having to quit their jobs, reduce their work hours, or losing their jobs because of SSc. |
|  | Dealing with employers | Experiencing distress caused by their employers not being understanding towards their situations. |
|  | Finances | Experiencing distress due to having a hard time paying their bills. |
|  | Affording health care | Experiencing distress caused by the expenses associated with specialist appointments and other SSc related health care costs. |
| **Changing Family Roles** | Talking about SSc with family members | Experiencing distress caused by having to discuss SSc with their families. |
|  | Feeling like a burden in your family | Experiencing distress due to feeling like they are burdens within their families. |
|  | Difficulty maintaining roles and responsibilities within one’s family | Experiencing distress caused by having to maintain their roles and responsibilities within their families. |
|  | Lack of family support | Experiencing distress due to not having support from their families. |
| **Social Interactions** | General distress related to social interactions and interpersonal relationships | Experiencing distress due to social interactions and interpersonal relationships. |
|  | Comments from strangers | Experiencing distress caused by comments from strangers about their SSc. |
|  | Feeling labeled by the disease | Experiencing distress caused by being defined and labeled by SSc. |
|  | Being treated differently because of SSc | Experiencing distress because they feel they are being treated differently because of their SSc diagnosis. |
|  | People not understanding the severity of the disease | Experiencing distress due to others underestimating or not understanding the severity of their SSc. |
|  | Pushing yourself too hard | Experiencing distress due to pushing themselves past their physical and psychological limits. |
|  | Feeling other people’s pity | Experiencing distress because they feel their friends or family members pity them. |
|  | Lying about how you feel | Experiencing distress because they feel they have to lie about or minimize the severity of their symptoms or their SSc. |
| **Navigating the Health Care System** | General problems with the health care system | Experiencing distress associated with problems within the health care system. |
|  | Communicating with doctor | Experiencing distress as a result of trying to communicate with their physicians. |
|  | Frequent referrals to specialists | Experiencing distress due to having multiple specialist appointments. |
|  | Discussing sexuality | Experiencing distress as a result of having to discuss sexuality, intimacy, or intercourse with their physicians, friends, families, or partners. |
|  | Communication between physician and specialists | Experiencing distress due to being responsible for maintaining communication between physicians and specialists about their SSc. |
